# Correlation of *in Vitro* Cytokine Responses with the Chemical Composition of Soil-Derived Particulate Matter

**DOI:** 10.1289/ehp.8360

**Published:** 2005-09-29

**Authors:** John M. Veranth, Tyler A. Moss, Judith C. Chow, Raed Labban, William K. Nichols, John C. Walton, John G. Watson, Garold S. Yost

**Affiliations:** 1 Department of Pharmacology and Toxicology, University of Utah, Salt Lake City, Utah, USA; 2 Atmospheric Sciences Division, Desert Research Institute, Reno, Nevada, USA; 3 Department of Chemical Engineering, University of Utah, Salt Lake City, Utah, USA; 4 Department of Civil and Environmental Engineering, University of Texas at El Paso, El Paso, Texas, USA

**Keywords:** air pollutants, BEAS-2B, carbonaceous aerosol, cell line, environmental analysis, fugitive dust, interleukin-6, interleukin-8, lung epithelial cells, Speciation Trends Network, thermal/optical reflectance

## Abstract

We treated human lung epithelial cells, type BEAS-2B, with 10–80 μg/cm^2^ of dust from soils and road surfaces in the western United States that contained particulate matter (PM) < 2.5 μm aerodynamic diameter. Cell viability and cytokine secretion responses were measured at 24 hr. Each dust sample is a complex mixture containing particles from different minerals mixed with biogenic and anthropogenic materials. We determined the particle chemical composition using methods based on the U.S. Environmental Protection Agency Speciation Trends Network (STN) and the National Park Service Interagency Monitoring of Protected Visual Environments (IMPROVE) network. The functionally defined carbon fractions reported by the ambient monitoring networks have not been widely used for toxicology studies. The soil-derived PM_2.5_ from different sites showed a wide range of potency for inducing the release of the proinflammatory cytokines interleukin-6 (IL-6) and IL-8 *in vitro*. Univariate regression and multivariate redundancy analysis were used to test for correlation of viability and cytokine release with the concentrations of 40 elements, 7 ions, and 8 carbon fractions. The particles showed positive correlation between IL-6 release and the elemental and pyrolyzable carbon fractions, and the strongest correlation involving crustal elements was between IL-6 release and the aluminum:silicon ratio. The observed correlations between low-volatility organic components of soil- and road-derived dusts and the cytokine release by BEAS-2B cells are relevant for investigation of mechanisms linking specific air pollution particle types with the initiating events leading to airway inflammation in sensitive populations.

Ambient particulate matter (PM) is a complex mixture containing primary particles derived from geological, biological, and combustion sources and the secondary aerosol formed by gas-to-particle conversion. The mass-based ambient air standards in the United States for particles < 10 μm (PM_10_) and < 2.5 μm (PM_2.5_) were justified by epidemiology studies [U.S. Environmental Protection Agency (EPA) 1996]; however, existing data are still inadequate to identify the pollution sources that are the most relevant to health effects in sensitive populations (Glinianaia et al. 2004; Health Effects Institute 2002; Schwartz 2004). Elucidating the toxicological mechanisms that link specific chemical and physical characteristics of inhaled particles with biological responses *in vivo* and *in vitro* is an active area of research. *In vitro* experiments involving cultured mammalian cells that are treated with various types of ambient and laboratory surrogate particles are an important technique for investigating the basic mechanisms of particle toxicology, as reviewed by Fubini et al. (1998). Commonly used cell types include the immortalized human lung cell lines BEAS-2B and A549, normal human bronchial epithelial cells, freshly harvested macrophages, and cocultures of macrophages with epithelial cells. Many *in vitro* studies have focused on the up-regulation and release of interleukin-6 (IL-6) and IL-8 by airway cells because of the role of these cytokines in the initiation and resolution of inflammation.

Cytokines are soluble peptides that are involved in many signal transduction pathways regulating cell growth, differentiation, and death, as well as recruitment of neutrophils, macrophages, and other mobile cells to specific sites (Driscoll 1999; Kelley 1990; Thèze 1999). Much of the recent *in vitro* work with lung cells exposed to environmental particles has used enzyme-linked immunosorbent assay (ELISA) to measure IL-6 (Becker et al. 2003; Hetland et al. 2000; Veronesi et al. 2003), IL-8 (Koyama et al. 2000; Smith et al. 2000; Stringer et al. 1996), and tumor necrosis factor-α (TNF-α) (Brown et al. 2003; Driscoll 2000; Smirnov et al. 1999). IL-6 and IL-8 have been studied as markers of inflammatory response both in isolated cell culture models and in the bronchial alveolar lavage fluid of animals (Driscoll 1999; Nelson and Martin 2000), providing a direct biomarker link between *in vitro* and *in vivo* studies. Increased levels of cytokines in lung lavage fluid, sputum, and blood have been associated with human diseases, including chronic obstructive pulmonary disease (Chung 2001) and asthma. A statistically significant increase in blood IL-6 was observed in 18 human subjects exposed to ambient air PM at 100–150 μg/m^3^ during a smoke pollution episode (van Eeden et al. 2001), indicating that IL-6 is a sensitive marker.

Wind- and vehicle-generated mineral dust from open land and roads is a major source of PM, especially in arid climates, and each dust sample is a complex mixture of different particle shapes and sizes that are derived from various geological minerals, biogenic debris, and anthropogenic materials deposited from the atmosphere. The ubiquitous nature of soil-derived dust in the air motivated this study. Schenker (2000) concluded that agricultural exposure to inorganic dusts has a plausible association with chronic bronchitis, interstitial fibrosis, and chronic obstructive pulmonary disease. Pope et al. (1999) reported that differences in the PM_10_–mortality association between nearby Utah communities could be explained by excluding wind-blown dust episodes. Studies in Spokane, Washington, found no correlation between ambient soil dust and mortality (Schwartz et al. 1999) or hospital emergency department visits for asthma (Claiborn et al. 2002).

A study of ambient samples of PM_10_ from three different zones of Mexico City used A549 and J774A.1 (mouse monocyte) cells and measured viability, apoptosis, and IL-6 release (Alfaro-Moreno et al. 2002; Osornio-Vargas et al. 2003). That study showed differences between the sites that appeared to be mediated by transition metals. Huang et al. (2003) correlated responses of BEAS-2B cells with the elemental composition and carbon fractions of ambient particles from Taiwan and found that the release of IL-8 correlated with chromium and manganese in the PM_1_ fraction. Mitkus (2004) used regression models in a study of several hundred ambient PM samples collected in Baltimore, Maryland, and concluded that multiple metals interacted with each other to cause *in vitro* cytokine release. A study of NHBE cell responses to ambient PM used principal component analysis to identify an elemental composition factor that correlated with IL-6 and IL-8 release (Becker et al. 2005), but the study did not analyze the carbon fractions. Principal components analysis was also used to test for relationships between gasoline and diesel engine exhaust samples and toxicity as measured by inflammation and tissue damage in rats (McDonald et al. 2004).

Crystalline silica is a potent inducer of cell responses (Rao et al. 2004), whereas titanium dioxide particles > 1 μm are often considered inert and used as a PM negative control (van Maanen et al. 1999). Mineral dust from stone quarries induced cytotoxicity and IL-6 release in A549 cells (Hetland et al. 2000) and in rat lung type 2 alveolar cells (Becher et al. 2001). Mineral composition other than quartz appeared to determine inflammatory potential of these mineral dusts (Schwarze et al. 2002), and Øvrevik et al. (2005) concluded that a particular mineral or element responsible for inducing cytokine release could not be identified. Hetland et al. (2004) concluded that road abrasion particles collected from a highway tunnel may be as potent as ambient air particles in inducing cytokine release, and multiple studies now show induction of lung cell inflammation by dusts that have been assumed to be benign.

Cell culture experiments have the advantage of using an inexpensive and convenient biological model that can be replicated in multiple laboratories, but an *in vitro* assay lacks the neurological signals and interactions between many different cell types that are important in a whole animal. The rank order of potency observed with an *in vitro* assay may differ from that observed *in vivo*. Seagrave et al. (2003) measured the responses of human lung epithelial cells and rat macrophages treated with the PM and semivolatile emission fractions from diesel and gasoline engines and found that the rank order of potency from the *in vitro* assays did not, in general, correspond with the previous rankings from *in vivo* comparisons of the same samples. A study with stone dust particles demonstrated a correlation between *in vitro* cytokine responses and the number of neutrophils in the bronchoalveolar lavage fluid of rats, but different rankings of potency of particles were found with *in vitro* assays using type 2 cells and alveolar macrophages (Becher et al. 2001). Results of *in vitro* assays are useful for linking particle types with the activation of signal transduction mechanisms leading to a cell response. The regulation of both IL-6 and IL-8 involves translocation of the transcription factor NF-κB (nuclear factor-κB) to the nucleus (Kennedy et al. 1998; Quay et al. 1998); however, there is evidence for other signaling pathways that regulate IL-6 and IL-8 independently.

In this study we tested the hypothesis that the variation in cytotoxicity and release of proinflammatory cytokines that is observed in a widely used *in vitro* lung cell line model (Frampton et al. 1999; Ghio et al. 1999; Kennedy et al. 1998; Veronesi et al. 2002) is correlated with the specific chemical species that are measured by the existing ambient monitoring network protocols. The U.S. National Park Service operates the Interagency Monitoring of Protected Visual Environments (IMPROVE) network, which has about 160 sites in or near national parks and large wilderness areas. The U.S. EPA operates the Speciation Trends Network (STN), which has approximately 200 monitoring sites, mostly in urban areas. These networks use similar sampling and analytical methods to measure elements and soluble ions, but differences exist in the protocol for analyzing the organic carbon (OC) and elemental carbon (EC) fractions (Chow et al. 2001). Immortalized human bronchial epithelial cells (type BEAS-2B) were treated with varying concentrations of PM_2.5_ dust derived from soils and road surfaces. Cell viability and the release of the cytokines IL-6 and IL-8 were measured, and standard statistical methods were used to test for correlations between variables.

## Materials and Methods

### Particles.

**Figure 1:**
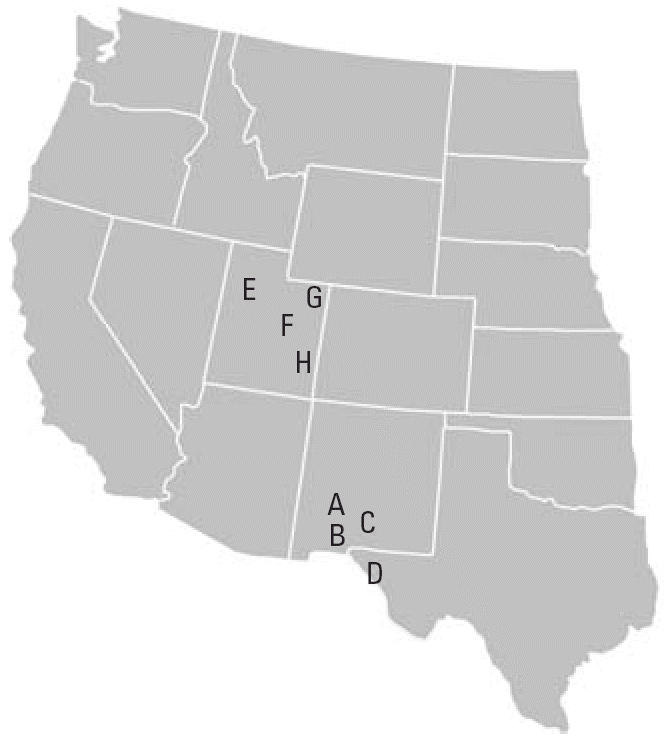
Sampling locations in the western United States.

**Figure 2:**
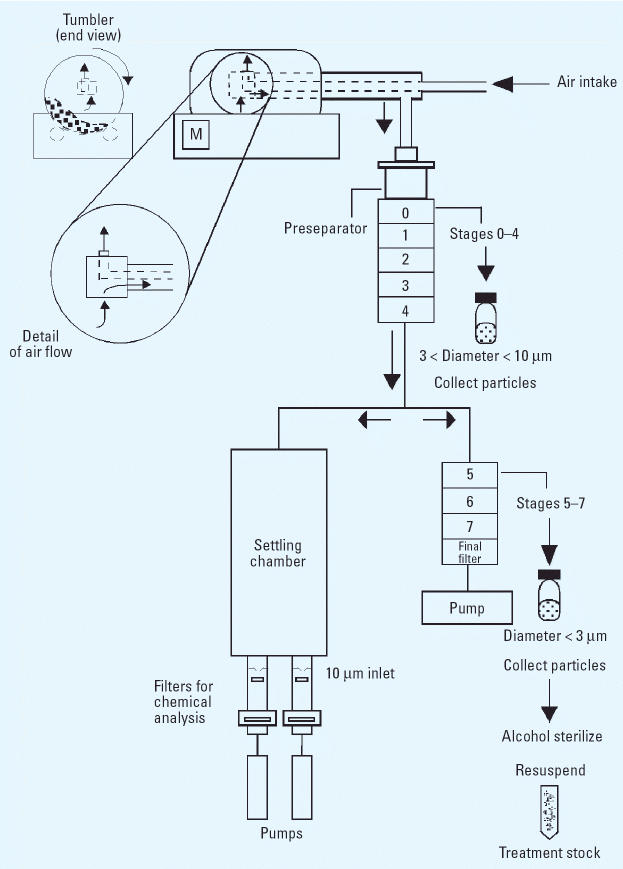
Laboratory resuspension and size fractionation of soil dusts were used to generate both bulk samples of particles for *in vitro* toxicology and filter samples for chemical analysis.

Samples of surface soil were collected from urban and rural sources of wind-blown dust, from unpaved roads and vehicle trails, and from the surfaces of paved streets after dust storms. The sampling sites are identified in Table 1 and Figure 1. Loose material was collected by making multiple traverses with a whisk broom to generate a composite sample, nominally 5 kg. A source apportionment study of fugitive dust from military training ranges in the western United States (Labban et al. 2004) provided many of the samples. The field samples were transported to the laboratory in sealed double bags. A PM_2.5_-enriched sample was extracted from the source material using a mechanical tumbler and a cascade impactor as described previously (Veranth et al. 2000) and illustrated in Figure 2. Filter samples for chemical analysis were also prepared using the tumbler with the resuspension chamber sampler.

Particle samples were weighed, resuspended in cell culture media, and diluted to appropriate concentration for the cell treatments. Previous experience indicated that adding soil-derived particles to cell culture media frequently resulted in the media becoming cloudy within 24 hr because of introduced viable organisms, but that either heat or alcohol pretreatment of the particles prevented this problem. For this study, the weighed particles were placed in a tube and wetted with a minimal amount of 70% ethyl alcohol, nominally 10 μL for 1–2 mg PM, and the alcohol was evaporated under vacuum to allow redeposition of any alcohol-soluble species on the particles before adding the cell culture medium. We previously reported the effect of aggressive heat treatment (150–550°C) and solvent leaching (three washings in large volumes of water or chloroform–methanol) treatment on the potency of three of the dusts used in this study (Veranth et al. 2004) and concluded that wetting with alcohol would introduce minimal artifacts. Also, a validation experiment with split portions of three archived filters showed that the alcohol treatment and drying did not cause statistically significant changes in any of the EC or OC fractions (data not shown). Lipopolysaccharide [LPS; 2,000 endotoxin units (EU)/mL] (*Pseudomonas aeruginosa*; Sigma Chemical Company, St. Louis, MO) and vanadyl sulfate (VOSO_4_; 80 μg/mL) (Alfa Aesar, Ward Hill, MA) were used as positive controls.

### Sample chemical analysis.

We determined elemental composition for the elements, from sodium through uranium, by X-ray fluorescence (Watson et al. 1998). The soluble ions were measured as follows: chloride, nitrate, phosphate, and sulfate ions by ion chromatography (Chow and Watson 1998); ammonium by automated colorimetry; and water-soluble sodium and potassium by atomic absorption. We determined eight carbon fractions based on desorption temperature using the thermal/optical reflectance (TOR) method following the IMPROVE protocol (Chow et al. 1993, 2001, 2004). OC fractions OC1, OC2, OC3, and OC4 correspond to the carbon released by heat treatment at 120°C, 250°C, 450°C, and 550°C in helium gas. Pyrolyzed carbon (OP) was defined by the carbon released while laser light reflectivity of the filter returned to the original value after 2% oxygen was added at 550°C. The EC fractions EC1, EC2, and EC3 correspond to carbon released at 550°C, 700°C, and 800°C in 2% oxygen/98% helium gas. The OC, EC, and total carbon (TC) values were calculated from the fractions. Carbonate carbon (CO_3_) was determined by acid pretreatment before TOR (thermal/optical reflectance) analysis (Chow and Watson 2002). The accuracy of the chemical speciation measurements was determined through calibration with traceable standards and replicate analysis.

### Cell culture.

BEAS-2B human bronchial epithelial cells, obtained from R. Devlin of the U.S. EPA, were maintained and passaged using the media and procedures originally developed by the U.S. EPA Human Studies Division and described in multiple publications (Frampton et al. 1999; Ghio et al. 1999; Kennedy et al. 1998; Veronesi et al. 2002). Cells were seeded in 24-well polystyrene plates (Costar; Fisher Scientific, Pittsburgh, PA) at a concentration of 35,000 cells/cm^2^, and KGM media (CC-3001, Cambrex BioProducts, Walkersville, MD) supplemented with the KGM-BulletKit (CC-3111) was replaced after 2 days. On the fourth day the cells were treated with particle concentrations of 10, 20, 40 and 80 μg/cm^2^, and the next day the cell culture media was harvested for the cytokine assays and cell viability was measured. Experiments were performed using triplicate wells for each treatment from two independent cell passages (i.e., *n* = 6).

We included negative and positive controls on each cell culture plate to monitor for any changes in cell phenotype. Throughout the study, the BEAS-2B cells in KGM media showed the previously observed low response to LPS and strong response to soluble vanadium compared with the untreated controls. No systematic change was observed over the experimental series, but absolute values of cytokine in the media varied more between passages than within a single culture plate. This passage variation affects the standard deviation.

### Cytokine ELISA.

We determined the concentrations of IL-6 and IL-8 in the cell culture media using sandwich ELISA assays. For IL-6, we used plates prepared with anti-human IL-6, biotin-conjugated anti-human IL-6, and avidin–horseradish peroxidase from eBioscience (San Diego, CA). All IL-6 values were quantified using a recombinant human IL-6 standard (R&D Systems, Minneapolis, MN). For the IL-8 ELISA, we used the DuoSet IL-8 development kit antibodies and standard (R&D Systems). Absorbances were read on a SpectraMax 250 plate reader with Softmax Pro (Molecular Devices, Sunnyvale, CA), and the concentrations were expressed as picograms per milliliter based on the standard curve obtained for each plate. ELISA data were analyzed using absolute concentrations based on the recombinant protein standard and fold increase over the control wells on the same culture plate.

### Viability.

We assessed cell viability using a tetrazolium dye assay (CCK-8, Dojindo Laboratories, Gaithersburg, MD) that measures mitochondrial activity. Viability relative to control was calculated by the absorbance at 450 nm corrected for the cell-free blank media absorbance. Previous experiments (Veranth et al. 2004) showed that inert particles remaining in the culture wells did not interfere with this assay; however, high concentrations of redox-active substances such as the vanadium IV ion can cause color change in cell-free media.

### Endotoxin.

We measured endotoxin using the chromogenic *Limulus* amebocyte lysate assay kit (QCL-1000; Cambrex BioProducts). Values are reported as EUs per milligram of dry particle sample.

### Statistics.

Data were analyzed using JMP software (SAS Institute Inc., Cary, NC). We evaluated significance using the probability of the observed trend being due to random variation as assessed by the *F*-test. A redundancy analysis was performed using CANOCO software, version 4.5 (Microcomputer Power, Ithaca, NY) (ter Braak and Prentice 1988; ehp0114-000341ter Braak and Smilauer 1998). Redundancy analysis created a set of orthogonal axes that were linear combinations of the independent variables, and regression analysis was then performed with the dependent variables versus the new axes.

## Results

**Figure 3:**
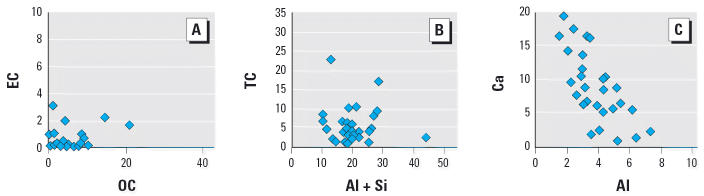
Examples illustrating the variation in sample composition (mass %) for the soil-derived dusts used in this study.

The soil- and road-derived dusts included a range of compositions as illustrated by the selected two-variable plots in Figure 3. The ratio of EC to OC in Figure 3A reflects the relative contribution of combustion soot versus organic material derived from biogenic activity and atmospheric photochemistry. The soils with high EC were from sites that had recent rangeland fires. The ratio of TC (EC + OC) to the sum of aluminum plus silicon (Figure 3B) is a measure of the contribution of inorganic minerals derived from the earth’s crust versus the fossil fuel and biomass contributions. The ratio of calcium to aluminum (Figure 3C) reflects differences in the geology of the source sites, for example, limestone compared with weathered clays.

### Cell assays with soil dusts.

**Figure 4:**
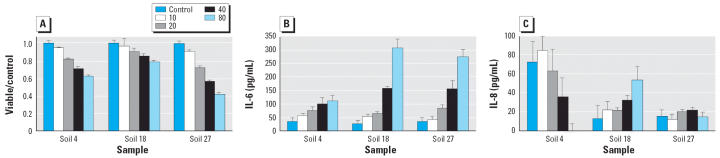
Typical concentration versus response data for viability, IL-6, and IL-8 [mean ± SD; *n* = 3 (one passage)] for three of the soil samples identified in Table 1. (*A*) Viable cell count divided by control. (*B*) IL-6 response; these and all other soil dusts showed increasing IL-6 with increasing particle concentration, in μg/cm^2^. (*C*) IL-8 response (varied between particle types: soil 4 showed the highest response at the 10 μg/cm^2^ particle concentration with decreasing cytokine response at higher concentrations; soil 18 showed an increasing response with increasing concentration; and soil 27 showed no trend). The difference in IL-8 control levels shown here was not unusual for passage to passage variation.

**Figure 5:**
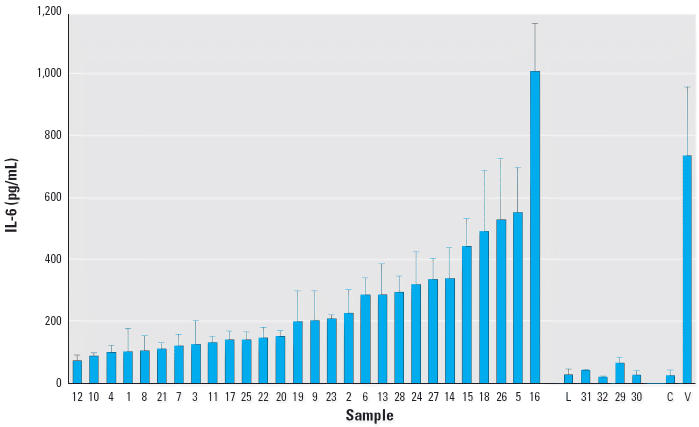
IL-6 release by BEAS-2B cells in response to treatment with 80 μg/cm^2^ of the indicated soil-derived particles (samples 1–28), nonsoil particles (L, samples 29–32), and controls (C, V): mean ± SD (*n* = 6: three cell culture wells times two independent passages). Sample numbers are identified in Table 1. Abbreviations: L, 2,000 EU/mL LPS; C, untreated control; V, 80 μg/mL soluble vanadium positive control. Some soil-derived dusts are as potent as an equal concentration of VOSO_4_, a major component of residual oil fly ash.

**Figure 6:**
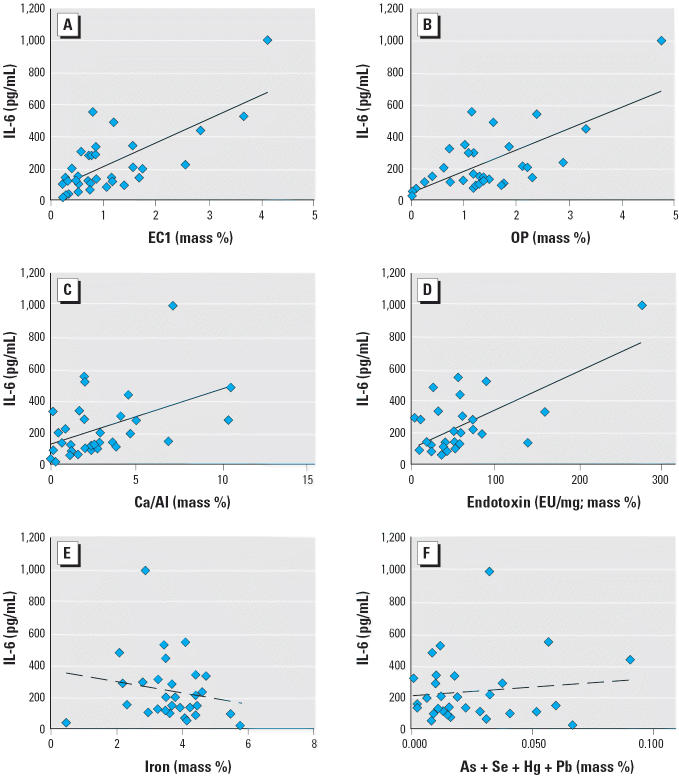
Univariate correlations of soil-derived dust composition variables with the concentration of IL-6 in the cell media at 24 hr. (*A*) EC1 (*R*^2^ = 0.50). (*B*) OP (*R*^2^ = 0.46). (*C*) Calcium/aluminum (*R*^2^ = 0.21). (*D*) Endotoxin (*R*^2^ = 0.43). (*E*) Iron (*R*^2^ = 0.03). (*F*) Arsenic + selenium + mercury + lead (*R*^2^ = 0.01).

**Figure 7:**
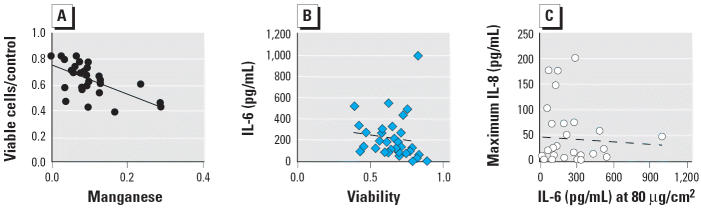
Univariate correlations between (*A*) cell viability at 24 hr and manganese concentration in soil-derived dust, (*B*) IL-6 and viability, and (*C*) IL-6 at 80 μg/cm^2^ and maximum IL-8. Cell viability at 24 hr was negatively correlated with Mn concentration in soil-derived dust (*A; R*^2^ = 0.37); IL-6 was not correlated with viability (*B; R*^2^ = 0.01); and IL-6 and IL-8 were not correlated (*C; R*^2^ = 0.003).

**Figure 8:**
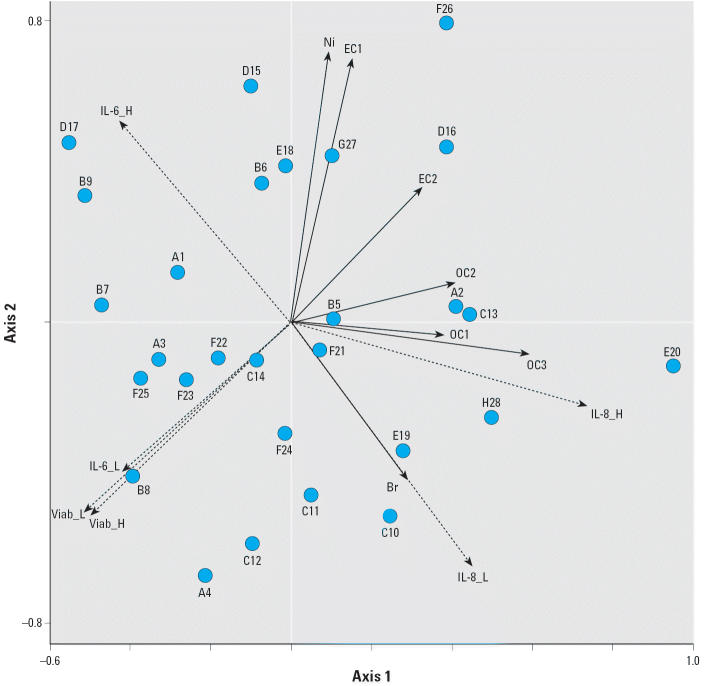
Results of a redundancy analysis using CANOCO software. Abbreviations: _H, high dose (80-μg/cm^2^); _L, low dose (10 μg/cm^2^); Viab, viability. The *x*- and *y*-axes are linear combinations of the most significant composition variables, which are shown by solid arrows (independent variables). Each circle indicates a sample (1–28) shown in locations A–H in Figure 1 and summarized in Table 1. Dashed arrows indicate the cell viability and cytokine release responses (dependent variables). The relationship of each independent variable to another variable is equal to the cosine of the angle between the arrows; a small angle indicates high positive correlation, and opposite directions indicate a negative correlation.

All the soil-derived PM_2.5_ induced a monotonic increase in IL-6 in the media with increasing particle concentration from 10 to 80 μg/cm^2^. Cell viability also decreased monotonically with increasing particle dose. Typical concentration–response results are presented in Figure 4. Figure 5 shows the IL-6 response of BEAS-2B cells treated with the 80 μg/cm^2^ dose of different soil-derived dusts (samples 1–28) compared to five nonsoil treatments (samples 29–32) and the untreated and positive controls. The soil-derived particles showed a wide range of potency, a necessary condition for doing a correlation study, and several of the soils induced an IL-6 response that was comparable with the response induced by soluble vanadium, the major active component in residual oil fly ash (Samet et al. 1997). The wide range of potency was a robust result, but the specific rank order of the samples was different at low and high PM concentration. Comparing the IL-6 response rank order lists at the 10 and 80 μg/cm^2^ concentrations, only five samples changed rank by more than 10 positions (i.e., by approximately one-third of the list). Figure 4 illustrates the cause of the change in rank. For soil 4, the IL-6 response at 10 μg/cm^2^ was moderate, but the slope with increasing particle concentration was small, and this soil was 26th in potency at high concentration but 5th in potency at low concentration. For soil 18, the IL-6 response slope with increasing particle concentration was much greater, and this soil had the same potency rank, 4th, at both concentrations. The data shown in Figures 4–8 were generated from 16 different cell culture experiments conducted over a 6-month period. The reproducibility of the *in vitro* cell response data was investigated to test for systematic variation in cytokine response or other factors that could confound the correlation analysis. The responses of the untreated control and of the LPS and vanadium positive controls showed random variation but no systematic change. As a further check, cells grown from frozen stocks several months after the main experiment were treated with a subset of the soil samples, and this confirmed that the observed differences between the potent and benign soil dusts were reproducible, but the specific rank order of samples for the replicate experiment differed slightly, as expected from the experimental variation indicated by the SD (error bars) in Figure 5.

The two coal fly ash samples (samples 29 and 30) and the calcined kaolin clay (sample 31) did not induce a strong IL-6 response compared with the soil-derived dusts. As expected, micrometer-sized titanium dioxide (sample 32) was a relatively benign particle. We tested for correlation between cytokine responses and various site categorical variables such as urban–rural, military–civilian, and road surface–open land. None of the tested categorical variables was statistically significant except for the difference between soil-derived and nonsoil samples.

Univariate correlations of the cell responses with the composition species are illustrated in Figures 6 and 7, and Table 2 lists all regression coefficients > 0.1. An *R*^2^ value of 0.1 corresponds roughly to a *p*-value of 0.05 using the *F*-test for the slope being different than zero. All univariate correlations were low (≤ 0.5), which indicates a large unexplained variation. The strongest positive correlations were between IL-6 and the concentration of EC1 and pyrolyzed OC (Figure 6A,B). All other EC and OC fractions had lower correlation coefficients. We observed a positive correlation between IL-6 and the calcium-to-aluminum ratio (Figure 6C), which suggests that high-calcium (alkaline) soils have a higher potency for IL-6 induction. Endotoxin is not measured by the STN and IMPROVE networks but was included in this correlation study because it is ubiquitous in ambient samples. We observed a correlation of IL-6 with endotoxin concentration (Figure 6D), but no statistically significant correlation with endotoxin remained when sample 16 was eliminated from the set. We observed no correlation between IL-6 release and the total concentration of iron, or the sum of the toxic metals arsenic, selenium, mercury, and lead (Figure 6E,F). The strongest negative correlation was between cell viability 24 hr after treatment and manganese concentration, shown in Figure 7A. The cytotoxicity of the particles and the release of IL-6 are not correlated (Figure 7B).

We observed release of IL-8 in response to some soil-derived particle treatments, but many of the concentrations of IL-8 for both control and treated cells were very low and near the limit of detection. All the soil dust particle treatments resulted in IL-8 levels that were low compared with the IL-8 induced by the soluble vanadium positive control. A positive univariate correlation was observed for IL-8 with carbonate carbon and EC3 (Table 2). The IL-8 response did not always increase with increasing particle dose over the range of 10–80 μg/cm^2^ (Figure 4). Some of the particle treatments showed the highest IL-8 release at the 10 μg/cm^2^ treatment concentration. In these cases, the decrease in IL-8 at higher particle concentrations was not explained by loss of cell viability. The observation of an increasing trend of IL-8 release with increasing particle concentration was positively correlated with the EC and OC fractions in the particles and is indicated by the IL-8 trend column in Table 2. The concentrations of IL-6 and IL-8 were not well correlated with each other (Figure 7C). The two coal fly ash materials and some soils, such as sample 28, induced high IL-8 release and low IL-6 release, whereas other soils, such as samples 16 and 5, induced high IL-6 but not IL-8.

### Multivariate analysis of responses to soil dust.

A major benefit of multivariate techniques is that they provide an overall picture of complex data sets and can reveal relationships that are difficult to perceive from univariate correlations. Figure 8 shows the relationship between the samples, independent variables (the concentrations of chemical species in the particles), and dependent variables (cell responses) based on multivariate redundancy analysis with CANOCO software. In this type of diagram, the independent variables and dependent variables are shown with the head located at the value of the correlation coefficient of the variable with each axis. The individual soil samples are indicated, and the sites are identified in Table 1. The relationship of each independent variable to another variable is equal to the cosine of the angle between the arrows. A small angle indicates high positive correlation, and opposite directions indicate a negative correlation. The directions of the orthogonal axes are not significant themselves, only the relationship between the variables and samples.

The axes are orthogonal linear combinations of the independent variables that were selected by the algorithm to explain the variance in the dependent variables. The seven independent variables included in the graph were sequentially selected as the most significant, all with *p* < 0.05 at time of inclusion in the model. The weighting of the normalized independent variables in forming each axis is equal to the reflection of the arrow for the variable on the axis. For example, axis 1 is positively associated with all of the indicated composition variables, and these variables, in order of increasing correlation coefficient, are nickel, EC1, bromine, EC2, OC2, OC3, and OC1. Axis 2 is positively associated with Ni, EC1, and EC2, negatively associated with Br, and weakly associated with OC1, OC2, and CO_3_. This redundancy analysis is significant at *p* = 0.0006 using an *F*-test. Axis 1 explains 28.2% of the variance of the cell responses, and axis 2 explains 21.8% of the variance. The low fraction of variance explained by the first two axes is consistent with the large unexplained variance indicated by the *R*^2^ values < 0.5 in the univariate analysis.

Increasing values on axis 1 are associated with increased IL-8 release, decreased viable cell count, and decreased IL-6 release at both the low (10 μg/cm^2^) and high (80 μg/cm^2^) particle treatment concentrations. Increasing values on axis 2 are associated with increasing IL-6 release at the high dose but decreasing IL-6 at the low dose and with decreases in cell viability and IL-8 release. Cell viability at both the low and high particle level are highly correlated with IL-6 release at the low treatment dose. IL-6 responses at the low and high particle treatment concentrations are uncorrelated, as indicated by the nearly perpendicular arrows (cosine ≈ 0), but this appears to be driven by a few samples that induced moderate IL-6 at low concentration and showed little additional increase in IL-6 response at higher concentrations. IL-6 response at the low concentration is negatively correlated with the concentration of the various carbon fractions, as indicated by arrows in opposite directions. At the high particle concentration the IL-6 response is partially correlated with both EC1 and Ni (≅ 50° angle, cosine > 0.5). The correlation between IL-6 and EC-1 was evident in the univariate correlations, but the relationship with Ni was not. The variation of Ni and EC1 are correlated, as indicated by the nearly parallel arrows, suggesting a common source for both. Combustion, for example, is a source for both Ni and EC. The IL-6 and IL-8 responses show a small negative correlation, as indicated by arrows in approximately opposite directions, and this is consistent with the univariate analysis in Figure 7C. In the multivariate analysis, IL-8 release at the 10 μg/cm^2^ dose was strongly correlated with Br, but this relationship was not evident in the univariate analysis. The Br may be a marker that is correlated with an unmeasured compound or emission source that is causing the response. Br was historically associated with leaded gasoline.

## Discussion

The established air monitoring networks are producing a long-term record of ambient aerosol composition, so it is worthwhile to apply the type of chemical speciation data produced by these networks to the analysis of laboratory toxicology study results. This is the first study to use a large number of soil-derived dusts to test whether the *in vitro* cytokine secretion responses in a widely used immortalized lung epithelial cell line treated with varying concentrations of particles were correlated with, or explained by, the specific chemical species, such as the functionally defined carbon fractions, measured using the STN and IMPROVE monitoring network protocols.

We observed a number of statistically significant correlations between chemical composition and IL-6 and IL-8 release by human lung cells. However, because of the multiple post hoc correlations, even a 1% confidence limit likely includes some type 1 error false positives. The major new insight from this study is that the strongest correlations were with EC and OC. Each of these TOR assay carbon fractions represents a complex mixture of compounds with similar vapor pressure and thermal stability, not a single molecular structure or group of related molecules. It is therefore unlikely that a TOR carbon fraction is precisely the true agonist in the *in vitro* assay. Rather, a correlation between these functionally defined carbon fractions and cytokine release suggests that the toxicologically relevant chemical compounds in soil-derived dust are highly correlated with the carbon fractions that are measured by the IMPROVE network protocol. For soil-derived particles, the correlation with carbon fractions is surprising because fugitive dust is dominated by inorganic material and typically contains < 10% TC (Labban et al. 2004). Thus, it is likely that some chemical species in the OC and EC fractions are highly inflammatory to lung cells. The correlation of IL-6 with the less volatile carbon fractions OC3, OP, and EC1 is consistent with previous results from our laboratory showing that heating soil-derived particles to 150°C had little effect on cytokine induction, and that heating the particles to 300°C or 550°C was required to remove the chemical compound or particle characteristic that induced the IL-6 response of BEAS-2B cells (Veranth et al. 2004). Ghio et al. (1996) reported that generation of oxidants by ambient particles correlated with the content of humic-like substances, again suggesting a role for the low-volatility, high-molecular-weight organic fraction in the induction of proinflammatory responses *in vitro*. Lipid peroxidation in BEAS-2B cells has also been reported to correlate with the OC fraction (Huang et al. 2003).

No strong associations were observed between IL-6 or IL-8 release and any of the redox-active transition metal concentrations as measured by X-ray fluorescence. A recent study of stone quarry dust with A549 cells also failed to find a correlation between cytokine release and elemental composition (Øvrevik et al. 2005). A plausible explanation is that the bioavailable metal fraction, not the total elemental concentration, is the toxicologically relevant variable. Previous studies with iron have shown that cell responses correlate with the amount of metal that is bioavailable under physiologically relevant pH and chelator conditions. Studies with coal fly ash (Ball et al. 2000), urban PM (Smith and Aust 1997), and asbestos (Lund and Aust 1992) have shown that iron mobilized by chelators correlates with the induction of ferritin synthesis by A549 cells and with DNA strand breaks in a cell-free assay for reactive oxygen species. Bioavailable iron and calcium in coal dust were found to correlate with regional differences in pneumoconiosis (Zhang et al. 2002).

It is possible that the currently used techniques for analyzing the composition of ambient particles samples are missing the chemical species that are most relevant for toxicology. Speculation as to the missed species includes bioavailable metal-containing compounds, anthropogenic organic compounds, biogenic organic material other than endotoxin, reactive molecules such as peroxides and stable free radicals adsorbed on the particle surface, and catalytically active mineral surface sites.

The strongest correlations we observed had an *R*^2^ value of about 0.5, which is higher than the single metal correlations reported by Mitkus (2004). The correlation coefficients between 0.1 and 0.5 in Table 2 support the hypothesis, advanced by Mitkus (2004), that multiple metals and other species interact to induce the cytokine responses. This study, as well as other *in vitro* cytokine release studies, shows considerable variation in the absolute values of the secreted protein for both control and treated conditions, and this cell culture variation confounds interpretation of results. Larger sample size is needed to maintain statistical power as treatment dose decreases because the responses at low dose approach control levels.

Airway epithelial cells can initiate and amplify airway inflammation by producing a number of proinflammatory mediators, and both *in vivo* and *in vitro* studies have shown that PM exposure results in increases of cytokines such as IL-6 and IL-8. Soil dust from wind erosion and vehicle travel contributes to the ambient PM mixture and is a plausible contributor to the epidemiological correlations between PM concentration and health effects.

## Conclusion

In this study we showed that some soil- and road-derived dusts induce secretion of the proinflammatory cytokines IL-6 and IL-8 by lung cells *in vitro* and that the potency is statistically associated with higher concentrations of the low-volatility OC and EC carbon fractions that are measured by the TOR assay. The observation that the strongest correlations were with the carbon fractions was unanticipated because soil-derived PM_2.5_, especially vehicle-generated road dust, is dominated by inorganic material. These *in vitro* results contribute to the long-term goal of integrating chemical speciation data for ambient PM mixtures with toxicology studies of specific pro-inflammatory cell signaling pathways. The total elemental concentrations measured by X-ray fluorescence were weakly correlated with cell responses, which is consistent with prior studies and suggests that additional chemical species should be quantified in future correlation studies. Given that increased IL-6 and IL-8 in lung fluid, sputum, and blood have been associated with clinical diseases, including chronic obstructive pulmonary disease and asthma, we believe that these *in vitro* results are applicable to lung inflammation and other human health effects. However, the range of *in vitro* particle concentrations used in this study was high compared with the airway surface average particle deposition that is calculated for ambient exposures. The correlations found in this study are most relevant for investigations of mechanisms linking exposure to specific particles and the initiating molecular events leading to proinflammatory signaling by tissues, and caution is needed in applying these *in vitro* results to real-world human exposures.

## Figures and Tables

**Table 1:** Source descriptions and sample identification codes.

Sample no.	Map location	Key	Site description	Surface use	Comment
1	A	A1	Rural	Dirt road	Interstate frontage road
2	A	A2	Rural	Grazing	Wind deposition area
3	A	A3	Rural	Road shoulder	Agriculture adjacent
4	A	A4	Suburban	Unpaved road	Area under development
5	B	B5	Rural	Grazing	Wind transport site in 2002
6	B	B6	Rural	Grazing	B5 site resampled in 2003
7	B	B7	Urban	Paved street	Elementary school adjacent
8	B	B8	Urban	River bed	Rio Grande
9	B	B9	Urban	Road shoulder	Horse stables adjacent
10	C	C10	Military	Dirt road	Main supply road
11	C	C11	Military	Dirt road	Sandy soil
12	C	C12	Military	Dirt road	After major dust storm
13	C	C13	Military	Training range	Recently burned
14	C	C14	Military	Training range	Mountain foothills
15	D	D15	Urban	Parking lot	Near smelter
16	D	D16	Urban	Paved street	Residential/commercial
17	D	D17	Urban	Paved street	Residential
18	E	E18	Military	Dirt road	Dust transport test site
19	E	E19	Rural	Dirt road	Remote site
20	E	E20	Rural	Dirt road	Playa
21	F	F21	Military	Training range	Small arms training
22	F	F22	Military	Training range	Small arms training
23	F	F23	Military	Training range	Smoke and obscurant training
24	F	F24	Military	Training range	Runoff impoundment
25	F	F25	Military	Training range	Artillery training
26	F	F26	Military	Training range	Recently burned
27	G	G27	Rural	Lake bed	Alpine recreation site
28	H	H28	Rural	Grazing	Mancos shale surface strata
29			Coal fly ash	Combustion	Utah bituminous coal
30			Coal fly ash	Combustion	New Mexico bituminous
31			Kaolin clay	Control particle	Ceramic material
32			Titanium dioxide	Control particle	1–2 μm particles
L			LPS	Positive control	
V			VOSO_4_	Positive control	Component of residual oil fly ash

Map locations are shown in Figure 1. The sample numbers and key are used in Figures 5 and 8, respectively.

**Table 2:** Summary of univariate correlations.

			IL-6				IL-8					
	Cell viability at 80 μg/cm^2^		10 μg/cm^2^		80 μg/cm^2^		10 μg/cm^2^		80 μg/cm^2^		Trend^a^	
	*R*^2^	*p*-Value	*R*^2^	*p*-Value	*R*^2^	*p*-Value	*R*^2^	*p*-Value	*R*^2^	*p*-Value	*R*^2^	*p*-Value
EU					0.43	#	0.13	*			0.21	*
K^+^									0.13	*		
Ca^2+^					0.21	*						
OC1			0.24	**								
OC2					0.22	**						
OC3					0.36	#					0.18	
OC4					0.31	**					0.26	**
OP					0.46	#					0.13	*
OC					0.43	#					0.19	*
EC1					0.50	#					0.26	**
EC2					0.13	*					0.31	**
EC3	0.16	*							0.13	*	0.30	**
EC											0.39	#
TC					0.43	#					0.27	**
CO_3_							0.18	*	0.27	**		
Mg							0.11		0.12	*		
Si					0.12	*	0.13	*	0.15	*		
P			0.23	**								
K	0.11	*										
Ca					0.21	*						
Cr											0.16	*
Mn	0.37	#							0.12			
Ni											0.27	**
Zn					0.31	**						
Se	0.13	*										
Br							0.16	*				
Sr									0.25	**	0.19	*
Hg	0.12	*										
Ca:Al					0.21	*						

For clarity, correlations with *R*^2^ < 0.1 are not shown.

a IL-8 trend indicates whether IL-8 concentration showed a positive correlation with treatment concentration over the range of 10–80 μg/cm^2^. Probability for slope different from zero by the *F*-test:

* 0.01 < *p* ≤ 0.05 (due to the multiple comparisons, some of these correlations are likely to be false positives);

** 0.001 < *p* ≤ 0.01;

# *p* ≤ 0.001.
